# Effects of the Insemination of Hydrogen Peroxide-Treated Epididymal Mouse Spermatozoa on γH2AX Repair and Embryo Development

**DOI:** 10.1371/journal.pone.0038742

**Published:** 2012-06-26

**Authors:** Jianfeng Xiao, Yanmei Liu, Zhiling Li, Yongcui Zhou, Hong Lin, Xiaoyan Wu, Man Chen, Wanfen Xiao

**Affiliations:** 1 Reproductive Center, The First Affiliated Hospital of Shantou University Medical College, Shantou University, Shantou, Guangdong, People’s Republic of China; 2 Reproductive Center, Women and Children Healthcare Hospital, Qinzhou, Guangxi, People’s Republic of China; Louisiana State University and A & M College, United States of America

## Abstract

**Background:**

Cryopreservation of human semen for assisted reproduction is complicated by cryodamage to spermatozoa caused by excessive reactive oxygen species (ROS) generation.

**Methods and Findings:**

We used exogenous ROS (H_2_O_2_) to simulate cryopreservation and examined DNA damage repair in embryos fertilized with sperm with H_2_O_2_-induced DNA damage. Sperm samples were collected from epididymis of adult male KM mice and treated with capacitation medium (containing 0, 0.1, 0.5 and 1 mM H_2_O_2_) or cryopreservation. The model of DNA-damaged sperm was based on sperm motility, viability and the expression of γH2AX, the DNA damage-repair marker. We examined fertility rate, development, cell cleavage, and γH2AX level in embryos fertilized with DNA-damaged sperm. Cryopreservation and 1-mM H_2_O_2_ treatment produced similar DNA damage. Most of the one- and two-cell embryos fertilized with DNA-damaged sperm showed a delay in cleavage before the blastocyst stage. Immunocytochemistry revealed γH2AX in the one- and four-cell embryos.

**Conclusions:**

γH2AX may be involved in repair of preimplantation embryos fertilized with oxygen-stressed spermatozoa.

## Introduction

Assisted reproduction centers, andrology laboratories and sperm banks cryopreserve human semen before assisted-reproduction treatment. Such cryopreservation provides patients with “fertility insurance” for intrauterine insemination, especially with cryopreserved donor semen, conventional *in vitro* fertilization or intracytoplasmic sperm injection [1]. However, during cryopreservation, spermatozoa are exposed to physical and chemical stress that results in adverse changes in membrane lipid composition, motility, viability and acrosome status of sperm [2], [3], [4]. These changes reduce the fertilization ability of human spermatozoa after cryopreservation [5], [6].

Cold shock during sperm cryopreservation is associated with oxidative stress and reactive oxygen species (ROS) generation [7]. ROS-induced damage to spermatozoa is mediated by oxidative attack of the bis-allylic methylene groups of sperm phospholipid-bound polyunsaturated fatty acids, thus leading to lipid peroxidation [8], [9]. The effects of lipid peroxidation include irreversible loss of motility, damage to sperm DNA or deficiencies in oocyte penetration and sperm–oocyte fusion [1], [10].

The marker of DNA damage is the formation of γH2AX focus, which is phosphorylated by ataxia telangiectasia mutated kinase. H2AX is one of the H2A variants with a conserved Ser-Gln-Glu motif at the C terminus; its phosphorylation plays a key role in DNA damage [11], [12]. γH2AX may promote recombination and conformational changes of chromatins, which may prevent the premature separation of broken ends, a function that would safeguard against harmful chromosome rearrangements [12].

Although numerous reports have described DNA damage to sperm caused by exogenous ROS (H_2_O_2_) or cryopreservation, only sperm motility variables and intracellular ROS and nitric oxide levels were examined [13], [14], [15]. The DNA damage to sperm needs to be repaired after fertilization to ensure correct genetic message transfer to progeny. Previous study showed that exogenous H_2_O_2_ increases intracellular ROS levels in spermatozoa [16].

Double-strand breaks are the most deleterious DNA lesions, which, if left unrepaired, may have severe consequences for cell survival, because they lead to chromosome aberrations, genomic instability, or cell death. The formation of DSBs activates many factors, including phosphorylation of the histone variant H2AX, producing γH2AX. Analysis of γH2AX expression can be used to detect the genotoxic effect of substances [11], for a repair marker of DNA damage. We aimed to examine embryos fertilized with sperm showing cryopreservation-similar DNA damage and DNA repair in terms of the organization and biological character of early embryos. We used an external source of ROS (H_2_O_2_) to simulate cryopreservation and observe the repair of DNA damage by the DNA-damage marker γH2AX in embryos fertilized with sperm with oxygen-induced DNA damage.

## Results

### DNA-damaged Spermatozoa Model Induced by H_2_O_2_

**Figure 1:**
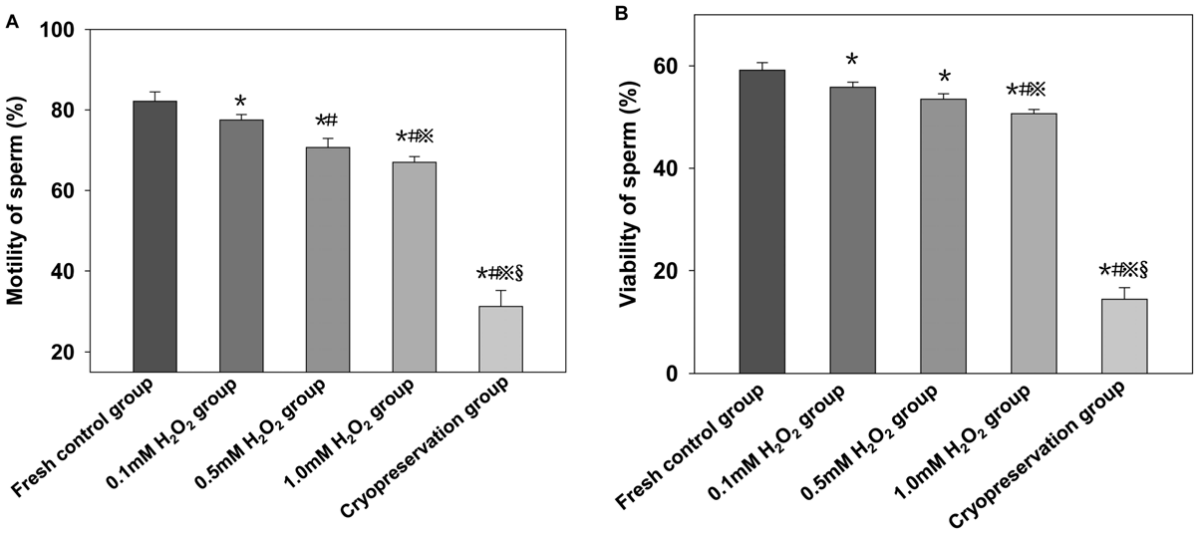
Motility of mouse spermatozoa (A) and viability of spermatozoa with different treatments (B). Data are mean±SD, *P<0.05 compared with fresh control group, # *P*<0.05 compared with 0.1 mM H_2_O_2_, ? *P*<0.05 compared with 0.5 mM H_2_O_2_, §*P*<0.05 compared with 1 mM H_2_O_2_.

Sperm was exposed to capacitation medium containing 0, 0.1, 0.5 or 1 mM H_2_O_2_. The percentage motility and viability of sperm was lower for cryopreserved than fresh sperm (*P*<0.05; Fig. 1). The percentage motility and viability was lower with cryopreservation than with 0.1, 0.5 and 1 mM H_2_O_2_ (*P*<0.05) and decreased with increasing H_2_O_2_.

### Induced DNA Damage in Sperm

**Figure 2:**
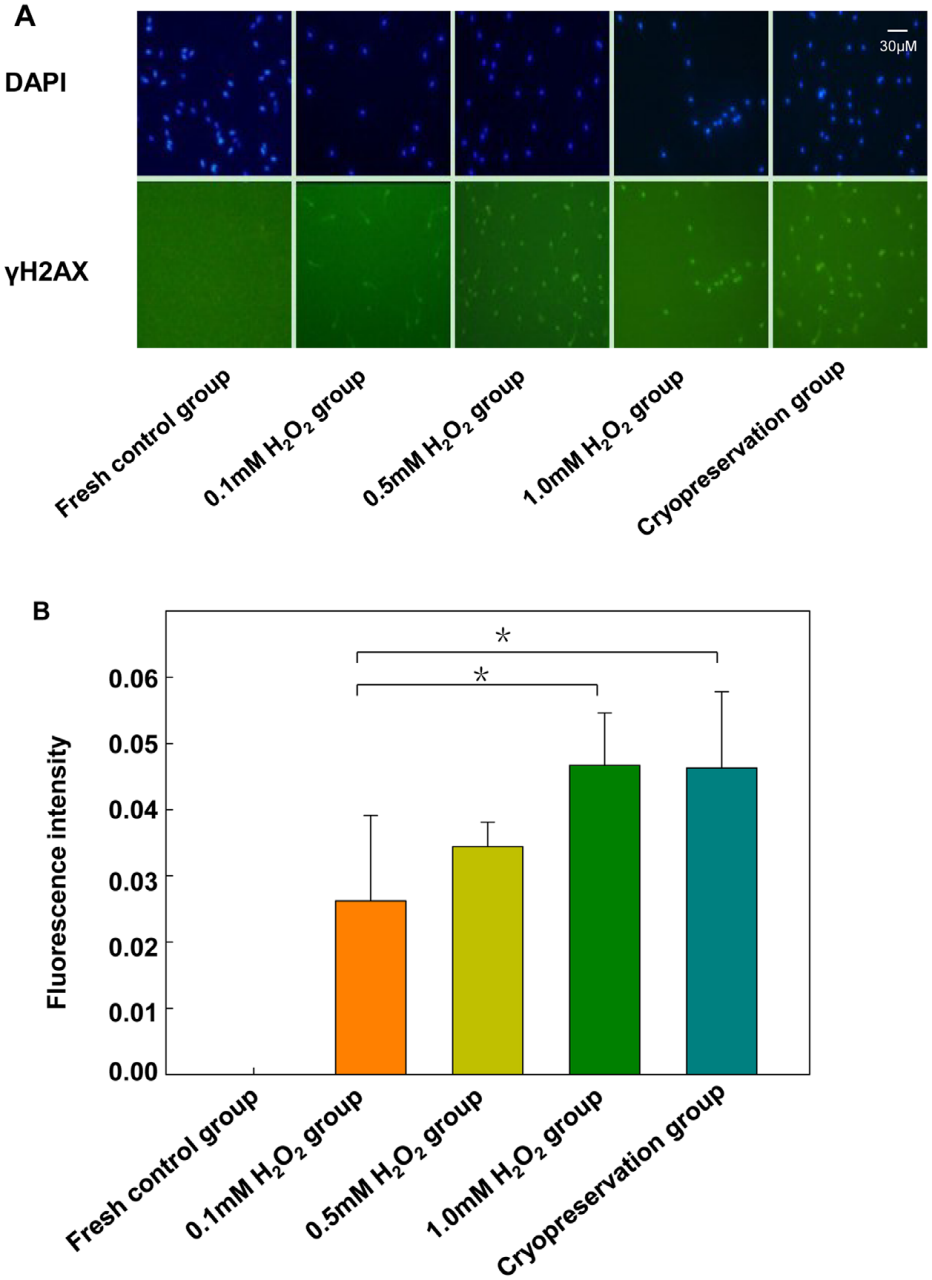
Fluorescence intensity of γH2AX in nuclei among fresh control, H_2_O_2_ and cryopreserved sperm (A) and quantification (B). Data are mean±SD, *P<0.05 compared with 0.1 mM H_2_O_2_. DAPI = staining of nuclei.

We examined the presence of the DNA-damage repair marker γH2AX in spermatozoa by immunocytochemistry. γH2AX expression was absent in fresh sperm but was present in cryopreserved sperm, and the level increased with increasing concentration of H_2_O_2_ (Fig. 2). The DNA damage, as determined by γH2AX level, was close with cryopreservation and 1-mM H_2_O_2_ treatment, so we chose 1 mM H_2_O_2_ as the DNA damage model induced by oxidative stress in the following experiments.

### Effect of H_2_O_2_ on Fertility and Embryo Development of Mice

Spermatozoa fertility and embryo development was similar with fresh spermatozoa and sperm treated with 1-mM H_2_O_2_ (*P*>0.05, Table 1).

**Table 1:** Fertilization *in vitro* and development of mouse embryos with fresh spermatozoa and sperm treated with 1-mM H_2_O_2_.

Treatment	Total eggs	Fertilized eggs (%)	Two-celled embryos (%)	Four-celled embryos (%)
Fresh spermatozoa	97	52 (53.6)	45 (86.5)	38 (84.4)
1-mM H_2_O_2_	106	51 (48.1)	42 (82.4)	34 (81.0)

### Effect of H_2_O_2_ on Cleavage of Embryos

No embryos had cleaved 12 h after insemination with fresh spermatozoa and sperm treated with 1 mM H_2_O_2_. At 16 h, the percentage of cleaved embryos was 69% and 58% for fresh spermatozoa and sperm treated with 1 mM H_2_O_2_, respectively, and was 87% and 73% at 36 h. The cleavage of one- to two-celled embryos did not change between 36 and 48 h for fresh spermatozoa but peaked at 40 h, at 82%, for sperm treated with 1 mM H_2_O_2_.

**Figure 3:**
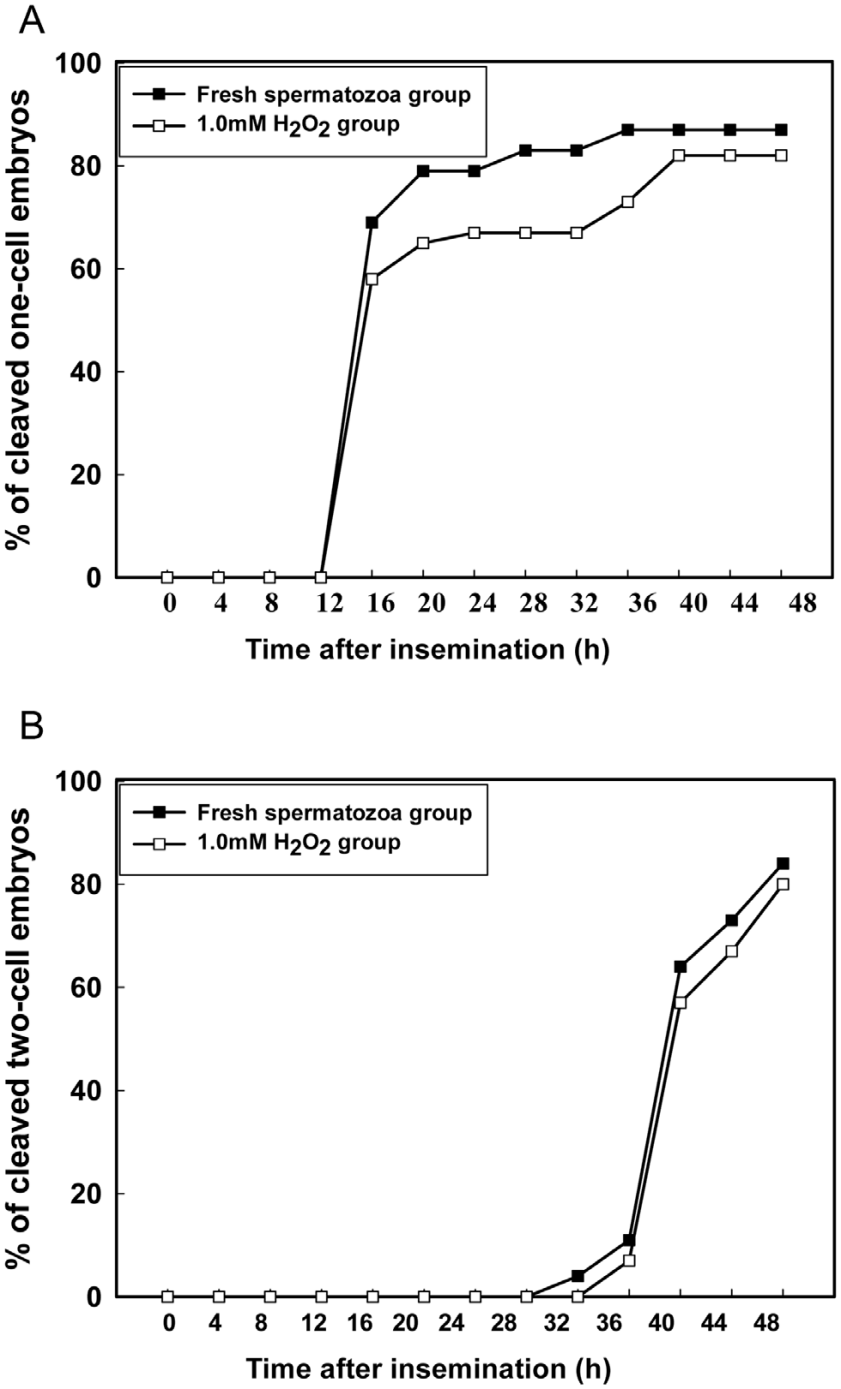
Delay in cleavage of one- and two-celled embryos after fertilization. Sperm was treated with 1 mM H_2_O_2_ and the time of embryo cleavage was examined. A: Cleavage rate of one- to two-celled embryos and B: two- to four-celled embryos.

At 32 h after insemination, 4% of four-celled embryos had cleaved with fresh spermatozoa. However, no embryos had cleaved with sperm treated with 1 mM H_2_O_2_. At 36 h, the percentage cleaved embryos was 11% and 7% for fresh spermatozoa and sperm treated with 1 mM H_2_O_2_, respectively, and at 48 hr, the percentage cleaved peaked at 84% and 80%, respectively. Thus, cleavage of one- and two-celled embryos was delayed in part in sperm treated with 1 mM H_2_O_2_ as compared with fresh spermatozoa after insemination (Fig. 3).

### Expression of γH2AX in Embryos Fertilized with H_2_O_2_-treated Sperm

**Figure 4:**
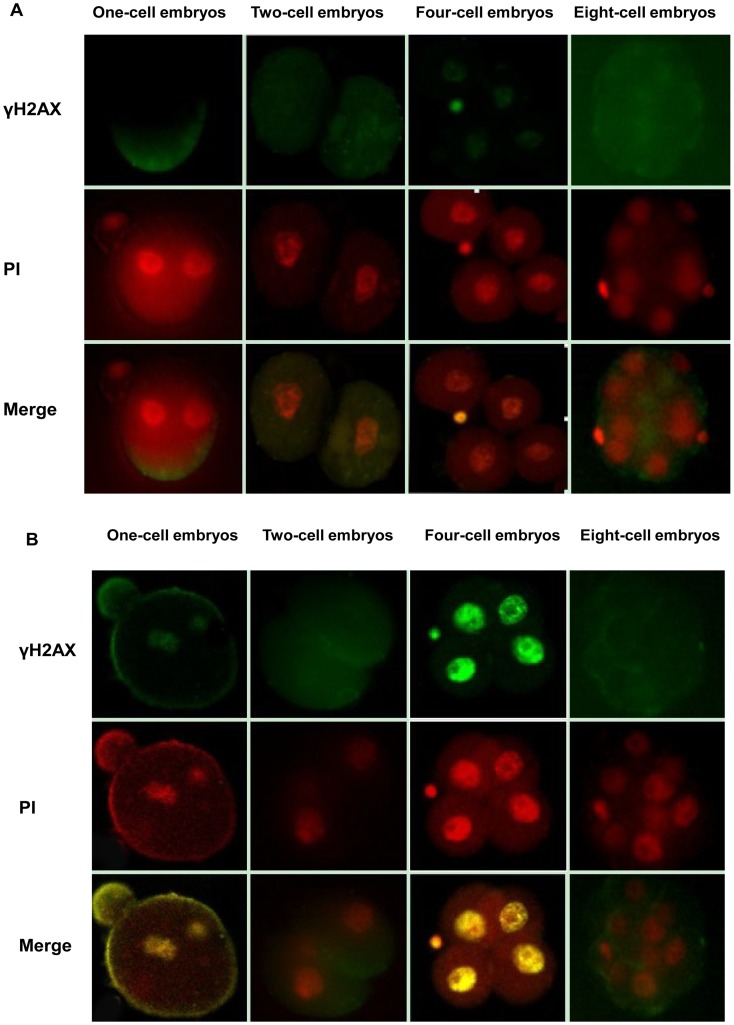
Immunohistochemistry of γH2AX expression in embryos. Changes in γ-H2AX expression in embryos fertilized by A: fresh sperm and B: sperm treated with H_2_O_2_. PI = propidium iodide staining.

We examined the presence of γH2AX in fertilized mouse embryos. In embryos fertilized with fresh spermatozoa, no embryos showed γH2AX expression. However, γH2AX expression was detected in one- and four-celled embryos fertilized with sperm treated with 1-mM H_2_O_2_ (Fig. 4). Therefore, the DNA repair mechanism was functional in one- and four-celled embryos fertilized with oxygen-stressed sperm.

## Discussion

Cryopreservation of human semen for assisted reproduction is complicated by cryodamage to spermatozoa caused by excess ROS generation. We used exogenous ROS (H_2_O_2_) to simulate cryopreservation and examined DNA damage repair in mouse embryos fertilized with mouse sperm with H_2_O_2_-induced DNA damage. Most of the one- and two-celled embryos fertilized with sperm treated with H_2_O_2_ showed a delay in cleavage before the blastocyst stage. Immunocytochemistry revealed the expresison of the DNA-repair marker γH2AX in the one- and four-celled embryos. γH2AX may be involved in DNA repair of preimplantation embryos fertilized with oxygen-stressed spermatozoa.

The cryopreservation of human semen plays an important role in the clinical management of male infertility. However, cryopreservation has been found to exacerbate damage to sperm, with reduced sperm motility and viability [1]. Vitality, chromatin normality, morphologic features, and membrane integrity of spermatozoa were severely altered by a freeze–thaw technique [17]. During the freeze–thaw process, excessive ROS was generated. Oxidative stress has long been implicated as major etiological factor in DNA damage in sperm.

ROS play a key role in the modulation of sperm function in the physiologic and pathologic environment. Physiologic concentrations of ROS are necessary to maintain normal sperm function. However, high levels can cause sperm dysfunction [18]. We found the percentage motility and viability of spermatozoa decreased significantly with cryopreservation, with no difference between fresh sperm and treatment with 0.1, 0.5 and 1 mM H_2_O_2_. However, DNA damage was close between cryopreservation and 1 mM H_2_O_2_ treatment, so we chose 1 mM H_2_O_2_ as a DNA-damage model induced by oxidative stress in the experiments.

Semen ROS are generated by spermatozoa (especially defective or immature sperm) and semen leukocytes [18], [19], [20], [21], [22]. In contrast to somatic cells, sperm are vulnerable to oxidative stress because of their unique membrane structures, as well as limited antioxidants or protective enzymes [23]. Spermatozoa and seminal plasma possess an antioxidant system of taurine, reduced glutathione (GSH), glutathione peroxidase (GSH-Px), catalase and superoxide dismutase to prevent oxidative damage [24]. However, semen selected by gradient centrifugation is necessary in assisted reproductive technology. Seminal plasma, sperm fragmentation and pathogenic microorganisms are cleared through assisted reproductive technology. Seminal plasma is the source of antioxidant systems, so sperm is in a superoxidative state after semen is selected. [25], [26].

We found no difference in fertilized egg rate and two- or four-celled embryos fertilized with fresh spermatozoa or 1-mM H_2_O_2_-treated sperm. In our previous study, the fertilized egg rate with cryopreserved sperm was lower than with fresh spermatozoa (*P*<0.05, unpublished data). The reasons for the loss of fertility are various, including factors affecting the proportion of survivors (e.g., cold shock susceptibility, cooling rate, diluent composition and osmotic stress) and factors influencing the functional status of survivors (e.g., membrane stability, oxidative damage, membrane receptor integrity, nuclear structure) [27]. The sperm motility rate and viability was lower with cryopreservation than with fresh sperm, with no difference between fresh sperm and 1-mM H_2_O_2_-treated sperm. However, the DNA damage was the same between cryopreserved and 1-mM H_2_O_2_–treated sperm on immunocytochemistry. DNA damage in sperm is negatively related with natural conception. The possibility of natural conception is zero with sperm DNA damge >30% [28]. The effects of sperm DNA damage on fertilization and embryonic development are still controversial. Sperm DNA damage increases the failure probability of *in vitro* fertilisation [29]. As well, DNA double-strand break is negatively related with fertilization rate [30]. Sperm DNA fragmentation seems to affect embryo post-implantation development in intracytoplasmic sperm injection: high sperm DNA fragmentation can compromise embryo viability, thus resulting in pregnancy loss [31].

We found no difference in fertilization rate or percentage of fertilized two- and four-celled embryos with fresh sperm and 1-mM H_2_O_2_-treated sperm. However, the time of cleavage to the two- and four-cell stage was delayed with 1-mM H_2_O_2_ treatment as compared with fresh sperm from 12 to 48 h after insemination. The cell checkpoint mechanism may play a role in embryo development. After sperm had been treated with 1 mM H_2_O_2_, phosphorylation of γH2AX was detected in one- and four-celled embryos, which suggests that the G2/M checkpoint and DNA repair mechanisms function in these embryos. The presence of γH2AX suggests that DNA repair mechanisms are functional in one- and four-celled embryos. γH2AX is required to maintain repair proteins at sites of DNA damage [32]. We did not observe phosphorylation of H2AX at one-, two-, four- and eight-celled embryos fertilized with fresh sperm.

Mature sperm cannot repair DNA damage. So the DNA damage must be repaired after fertilization to ensure the integrity of the DNA transferred to offspring [33]. The zygote mainly generates non-translated transcripts [34], [35]. In the mouse, transcription becomes fully functional during zygotic gene activation (ZGA) in the G2-phase of two-celled embryos [36], [37]. Hence, the mRNA and protein stored in the maternal gamete, the oocyte, must provide the zygote with the necessary molecules to perform all basic cellular functions until ZGA [38]. We observed this process indirectly. The γH2AX foci appeared in one-celled embryos and disappeared in two-celled embryos, then reappeared in four-celled embryos. The G2/M checkpoint mechanism seems to function insufficiently in one-celled embryos, but two-celled embryos are the stage of the maternal genome transforming into a zygotic genome. Perhaps the DNA damage caused by 1 mM H_2_O_2_ was repaired in part before ZGA. However, other DNA damage was not repaired, so the DNA damage was not enough to begin embryonic DNA damage repair. The unrepaired DNA damage signal was amplifed during embryonic development and activated the repair pathway after ZGA. The cleavage delay of the one-celled embryos to the two-celled stage and the two-celled embryos to the four-celled stage suggested that the G2/M checkpoint mechanism is operative.

### Conclusions

The repair of DNA damage that originates from oxidative-stressed sperm after fertilization depends on the mRNA and protein stored in the maternal gamete. The DNA damage caused by 1-mM H_2_O_2_ in sperm was repaired in part before ZGA. Other DNA damage is repaired during the early pre-zygotic stage of life when DNA repair pathways are activated because we found γH2AX expression in four-celled embryos fertilized with oxygen-stressed sperm.

## Materials and Methods

### Material and Reagents

All chemicals were of analytical grade. The mouse antibody for anti-phospho-histone H2AX (γH2AX; Ser 139) was from Millipore (USA) and goat anti-mouse Alexa Fluor 488 secondary antibody was from Invitrogen (Carlsbad, CA, USA). Human tubal fluid-HEPES (HTF-HEPES) was from SAGE (USA). Pancreatin was from Sigma (St. Louis, MO, USA). Phosphate buffered saline (PBS) was prepared in milli-Q water: 136.9 mM NaCl, 2.7 mM KCl, 0.9 mM CaCl_2_, 0.5 mM MgCl_2_, 7 mM Na_2_HPO_4_, 1.25 mM NaH_2_PO_4_, 1.5 mM KH_2_PO_4_, and 1 g/l PVA (290 mOsm/kg; pH 7.2). R18S3 was prepared with 1.8 g skim milk powder soluted into milli-Q water for 6% solution, then centrifuged at 4°C 15,000 g for 1 h. For 20 ml supernatant, 12.5 ml milli-Q water and 7.2 g raffinose was added, then milli-Q water was added to 40 ml; the mixture was agitated to solute raffinose, filtered, subpackaged and stored at –20°C. Pancreatin solution was prepared by the addition of 0.1% pancreatin to PBS, adjusting pH to 3.0 with HCl, filtering, subpackaging and storage at 4°C. Sperm capacitation liquid was prepared by adding 1.5% bovine serum albumin (BSA) to HTF solution. For fertilization liquid, 0.4% BSA was added to HTF solution. For embryo culture medium, 0.4% HTF and 10% fetal bovine serum was added to HTF solution. H_2_O_2_ sperm capacitation liquid was prepared by adding H_2_O_2_ to a final concentration of 0.1, 0.5 and 1 mM. T-PBS involved adding 0.05% Tween20 to PBS. Stationary liquid involved adding 4% paraformaldehyde to PBS. Membrane liquid was prepared by adding 0.2% Triton X-100 to PBS. Blocking buffer was prepared by adding 0.05% Tween20, 3% BSA and 10% goat serum to PBS. Sperm capacitation liquid, fertilization liquid and embryo culture medium were placed at 37°C in a 5% CO_2_ incubator for 4 h.

### Epididymal Sperm

Adult male KM mice (4–6 weeks old) **were** obtained from the animal center of Shantou University Medical College and treated in compliance with The Guide for the Care of Use of Laboratory Animals by the US National Institutes of Health (NIH Publication No. 85–23, revised 1996) and the rules of the National Animal Protection of China. All experimental protocols were approved by the Laboratory Animal Ethics Committee of our institution (SUMC2011-107). The study was approved by the Institutional Animal Care and Use Committee of Shantou University Medical College. Sperm was obtained from the caudae epididymis of mice and exposed to capacitation medium (containing 0, 0.1, 0.5 and 1 mM H_2_O_2_) at 37? in a 5% CO_2_ incubator for 1.5 h.

### Semen Cryopreservation

The cryoprotectant was thawed, mixed, and added to 35-mm culture dishes and warmed at 37°C in a 5% CO_2_ incubator. Cauda epididymis tissue was put in 1-ml warming cryoprotectant and extruded slightly to ensure no sperm. Spermatozoa were collected from the cauda epididymis and cultured at 37°C in a 5% CO_2_ incubator for 10 to 15 min. Sperm variables (density, motility rate and vitality) were recorded by light microscopy (×400 magnification). Spermatozoa were mixed with cryoprotectant and 100 µl was packed into 1.8-ml cryovials (Nalge Co., Rochester, New York) stored in a refrigerator at 4°C for 30 min. The samples were then frozen as follows: aliquots were suspended in liquid nitrogen vapor (8–10 cm above the level of liquid nitrogen at –80°C) for 10 min, plunged into liquid nitrogen (–196°C) completely, and stored in liquid nitrogen until use [1].

### Semen Analysis

Sperm motility was assessed by phase-contrast microscopy (100× magnification) with a warm stage maintained at 37°C. A 5-µl drop of semen was wet-mounted on a microscope slide and covered with a coverslip. Sperm motility was estimated in 3 different microscopy fields for each semen sample. The mean of the 3 successive estimations was recorded as the final motility score [24].

### Collection and Culture of Oocytes and Embryos

Fully grown germinal vesicle oocytes were collected from the ovaries of mice 13 to 15 h after injecting 10 IU human chorionic gonadotropin and 10 IU of the mate’s serum gonadotropin ahead 48 h. Cumulus oocytes were collected in PBS, then moved to fertilization liquid with use of a pipette and washed. The oocytes were imseminated with spermatozoa that had been preincubated for 1.5 h in sperm capacitation liquid. At 4 h after insemination in HTF medium, the embryos were washed 3 to 5 times, then incubated.

### Immunocytochemistry

A 5-µl sperm suspension was placed on slides, covered with polylysine and fixed with 4% paraformaldehyde for 15 min, washed with T-PBS 3 times, permeabilized with 0.2% Triton X-100 at room temperature for 30 min, washed with T-PBS 3 times, covered with goat serum working solution at room temperature for 2 h, incubated with primary antibody anti-γH2AX (1∶1000 dilution) for at 4°C for 12 to 15 h, then with secondary FITC-conjugated goat anti-mouse IgG antibody (1∶500; Santa Cruz Biotechnology, Santa Cruz, CA, USA) at 37°C for 1 h. The immunostained sperm were washed with T-PBS, counterstained with 4′,6-diamidino-2-phenylindole (DAPI), and observed under an Olympus FluoView FV1000 confocal microscope (Olympus Inc., Japan).

Embryos were washed with PBS and the zona pellucida was removed by digestion with pancreatin (0.1%) for 30 s, then embryos were fixed with 4% paraformaldehyde in T-PBS for 30 min, permeabilized for 30 min at room temperature with T-PBS containing 0.1 Triton X-100 and 3 mg/ml BSA, stained with anti-phospho-γH2AX antibody for 1 h at 37°C, then secondary FITC-conjugated antibody. The immunostained embryos were washed with T-PBS containing 0.1% Triton X, counterstained with propidium iodide, and observed under an Olympus FluoView FV1000 confocal microscope.

### Stastistical Analysis

Results are expressed as mean±SD. Data analysis involved use of SPSS 13.0 (SPSS Inc, Chicago, IL). Comparisons between 2 groups involved chi-square test and more than 2 groups one-way ANOVA. *P*<0.05 was considered statistically significant.
